# College Changes

**Published:** 1878-07

**Authors:** 


*College Changes.*—Dr. B. F. Coy, retires from the chair of Dental Surgery, Maryland Dental College, his place being filled by Dr. R. B. Donaldson, of Washington, D. C.; and Dr. S. M. Field, as Professor of Chemistry, is succeeded by Dr. R. Finley Hunt, of the same city. H.

